# An Improved Red-Billed Blue Magpie Optimization Algorithm for 3D UAV Path Planning in Complex Terrain

**DOI:** 10.3390/biomimetics11010043

**Published:** 2026-01-06

**Authors:** Yong Xu, Ning Xue, Yi Zhang

**Affiliations:** College of Electrical and Computer Science, Jilin Jianzhu University, Changchun 130119, China

**Keywords:** Red-Billed Blue Magpie Optimizer, UAV path planning, chaotic mapping, dynamic parameter adjustment, elite disturbance mechanism

## Abstract

This paper presents the Circle-Mapping Transition and Weighted Red-Billed Blue Magpie Optimizer (CTWRBMO), designed to address significant challenges in 3D path planning for drones. Although the original Red-Billed Blue Magpie Optimizer (RBMO) algorithm features a simple structure, few parameters, and strong local search capability, making it well-suited for UAV path optimization, it suffers from insufficient population diversity, limited global search ability, and a tendency to fall into local optima in complex high-dimensional scenarios. To overcome these limitations, four enhancement strategies are introduced. Firstly, the Circle chaotic mapping strategy leverages the randomness and ergodicity of chaotic sequences to generate an initial population that is uniformly distributed. This enhancement improves population diversity from the beginning and provides a solid foundation for global optimization. Secondly, the ε parameter is dynamically adjusted to prioritize local refinement during the early stages of optimization. This adjustment enables rapid convergence toward potentially optimal areas. This parameter increases to enhance global search capabilities as the algorithm progresses, thereby broadening the optimization space and achieving a dynamic equilibrium. Additionally, a nonlinear dynamic weighting factor (wd) is incorporated into the position update formula. The algorithm’s ability to escape local optima is significantly improved by dynamically altering the weight ratio between historical optimal positions and the current position. Furthermore, an elite perturbation mechanism based on individual neighborhoods is implemented to generate candidate solutions using local information. This mechanism enhances the algorithm’s local exploration capabilities and improves the stability of preserving optimal solutions, supported by a greedy criterion for optimal retention. Experimental results show that the CTWRBMO algorithm significantly outperforms comparison algorithms in terms of optimization accuracy and convergence speed, demonstrating exceptional global optimization capabilities. Additional applications in UAV 3D path planning simulations evaluated paths based on length, threat avoidance efficiency, and smoothness. The results indicate that paths planned using CTWRBMO are shorter, safer, and smoother compared to those generated by the Harrier Hawks Optimization (HHO), African Vulture Optimization Algorithm (AVOA), Artificial Bee Colony (ABC) Algorithm, and the traditional Magpie Algorithm, effectively meeting practical engineering requirements for UAV 3D path planning.

## 1. Introduction

Uncrewed aerial vehicles (UAVs) have rapidly evolved into an essential component of general aviation, showcasing applications in precision agriculture, logistics delivery, emergency response, disaster warning, aerial photography, and more. Two critical technologies that facilitate autonomous drone flight are path planning and obstacle avoidance. Any static or dynamic object can act as an obstacle during a drone’s flight, potentially causing damage to the aircraft or even leading to a crash in a three-dimensional environment. In addition to external obstacles, UAVs face constraints related to their own flight characteristics during path planning, which include flight altitude, turn angles, and maximum and minimum flight distances. Therefore, effective UAV path planning must not only focus on avoiding obstacles but also consider these inherent limitations of the vehicle. These factors significantly influence the efficiency and safety of mission execution. Path planning approaches for UAVs can generally be categorized into two main groups: classical algorithms and swarm-intelligence-based optimization techniques. Classical algorithms have been widely used for route computation, such as A* [1,2], Dijkstra’s algorithm [3], and genetic algorithms [4]. However, these methods often require considerable computational resources and memory when deployed in large-scale or high-dimensional environments. Researchers have begun to explore swarm intelligence optimization algorithms, including the Sparrow Search Algorithm [5], the Grey Wolf Optimizer [6], and the Artificial Bee Colony [7]. These algorithms mimic natural behaviors, such as foraging and mating, demonstrating strong global search capabilities and adaptability. Reference [8] integrated a bidirectional search strategy to enhance pheromone updating and next-node selection, effectively reducing the risk of premature convergence. Reference [9] incorporated genetic operations into Particle Swarm Optimization (PSO) to accelerate population convergence speed. Reference [10] utilized chaotic mapping during initialization and introduced dynamic boundary reverse learning to improve solution diversity and prevent local stagnation. Reference [11] introduced the Cauchy distribution for perturbation and adopted the greedy strategy to retain the better solutions, enhancing the escape ability, accelerating the convergence speed in the later stage, and improving the convergence accuracy of the algorithm. Reference [12] adopted an improved reverse learning strategy to expand the search space, enhancing the algorithm’s exploration ability for high-quality solutions, avoiding missing potential optimal paths. In Reference [13], the African vulture optimization algorithm integrates the discovery and vigilance mechanisms of the sparrow algorithm to balance exploration and exploitation, improving the algorithm’s early global search ability. Other metaheuristic algorithms, such as the Whale Optimization Algorithm [14], Dung Beetle Optimization [15], and Butterfly Optimization Algorithm [16], have also been applied to UAV path planning.

RBMO [17] is a recently developed swarm intelligence algorithm inspired by the cooperative foraging, predation, and food-hoarding behaviors of red-billed blue magpies. Benefiting from its cooperative population-based search mechanism, RBMO facilitates effective information sharing among individuals and has demonstrated promising performance in continuous optimization problems. Such characteristics are particularly relevant to three-dimensional UAV path planning, where the optimization variables are continuous, and the objective landscape is highly nonlinear due to terrain variation and obstacle distribution.

Nevertheless, the canonical RBMO exhibits several limitations when extended to strongly constrained and high-dimensional optimization scenarios. In particular, limited population diversity during the early search stage may reduce exploration capability, while the absence of adaptive regulation in the search dynamics can result in premature convergence and insufficient global exploration. In complex UAV path planning environments involving terrain constraints, obstacles, no-fly zones, and vehicle kinematics, these shortcomings may lead to infeasible trajectories or suboptimal flight paths.

Despite these limitations, RBMO was deliberately selected as the foundation of this study due to its flexible algorithmic structure, explicit behavioral mechanisms, and established effectiveness in continuous optimization. Compared with more rigid optimization frameworks, RBMO provides a suitable baseline for systematic enhancement, allowing targeted modifications to be integrated while preserving its inherent cooperative search advantages. This makes RBMO a meaningful candidate for adaptation to multi-constraint three-dimensional UAV path planning.

To overcome the aforementioned deficiencies, this study extends RBMO by incorporating several complementary strategies aimed at enhancing population diversity, strengthening global exploration, and improving convergence stability under complex constraints. The primary objective is to retain the intrinsic strengths of RBMO while significantly improving its robustness and solution quality in challenging and constrained UAV path planning environments.

## 2. Red-Billed Blue Magpie Optimization Algorithm

The RBMO algorithm is a metaheuristic approach inspired by the natural behaviors of red-billed blue magpies, including foraging, pursuing, striking, and storing food. Its optimization mechanism can be conceptually divided into three sequential stages: prey exploration, prey capture, and food preservation.

### 2.1. Prey Searching

Red-billed blue magpies typically search for food either in small groups (2 to 5 individuals) or large flocks (more than 10 individuals). To simulate their search strategies under different group sizes, Equation (1) is used to model the small-group searching behavior, while Equation (2) represents the large-flock searching behavior.

(1) $$X^{i}\left(t+1\right)=X^{i}\left(t\right)+\left(\frac{1}{p}\times \sum_{m=1}^{p}X^{m}\left(t\right)-X^{\mathrm{rs}}\left(t\right)\right)\times {\mathrm{Rand}}_{2}$$

(2) $$X^{i}\left(t+1\right)=X^{i}\left(t\right)+\left(\frac{1}{q}\times \sum_{m=1}^{q}X^{m}\left(t\right)-X^{\mathrm{rs}}\left(t\right)\right)\times {\mathrm{Rand}}_{3}$$

*t* denotes the current iteration number; p represents a randomly selected subgroup of 2–5 red-billed blue magpies from the entire population, while q denotes a group consisting of more than 10 individuals randomly chosen from the same population. The symbol $X^{m}\left(t\right)$ refers to the position of the m-th red-billed blue magpie randomly selected at iteration t, and $X^{\mathrm{rs}}\left(t\right)$ represents the position of a randomly chosen individual in the current iteration.

### 2.2. Prey Attacking

The red-billed blue magpies primarily target small prey or plants during the prey-attacking phase when acting in small groups. This behavioral pattern can be represented by Equation (4). When foraging in large flocks, the magpies tend to attack larger prey such as big insects or small vertebrates, which is described by Equation (5).

(3) $$CF={\left(1-\left(\frac{t}{T}\right)\right)}^{2\times \frac{t}{T}}$$

(4) $$X^{i}\left(t+1\right)=X^{\mathrm{food}}\left(t\right)+\mathrm{CF}\times \left(\frac{1}{p}\times \sum_{m=1}^{p}X^{m}\left(t\right){-X}^{i}\left(t\right)\right)\times {\mathrm{Randn}}_{1}$$

(5) $$X^{i}\left(t+1\right)=X^{\mathrm{food}}\left(t\right)+\mathrm{CF}\times \left(\frac{1}{q}\times \sum_{m=1}^{q}X^{m}\left(t\right)-X^{i}\left(t\right)\right)\times {\mathrm{Randn}}_{2}$$

Here, $X^{\mathrm{food}}\left(t\right)$ denotes the current global best position, while ${\mathrm{Randn}}_{1}$ and ${\mathrm{Randn}}_{2}$ represent normally distributed random numbers with a mean of 0 and a standard deviation of 1.

### 2.3. Food Storage

The Red-billed Blue Magpie also stores excess food in hidden places such as tree holes for future use in addition to searching for food and attacking prey. During this process, information about potential solutions is preserved, which assists individuals in finding the global optimal solution, as expressed in Equation (6).

(6) $$X^{i}\left(t+1\right)=\left\{\begin{array}{c} X^{i}\left(t\right)\;\;if\;{\;\mathrm{fitness}}_{\mathrm{old}}^{i}>{\mathrm{fitness}}_{\mathrm{new}}^{i}\\ X^{i}\left(t+1\right)\;\;\;\;\;\;\;\;\;\;\;\;\;\;\;\;\;\;\;\;\;\;\;\;\;\;\;\;\;\;\;\;\;else \end{array}\right.$$

The terms ${\mathrm{fitness}}_{\mathrm{old}}^{i}$ and ${\mathrm{fitness}}_{\mathrm{new}}^{i}$ represent the fitness values of the i-th Red-billed Blue Magpie before and after the position update, respectively.

The following pseudocode (Algorithm 1) illustrates the detailed procedure.
**Algorithm 1** The RBMO
1:**Begin**2:Initialize the relevant parameters (T, N, ε, etc.)3:     **while** t < T4:            Calculate the fitness of each search agent5:            Update the best solution6:            **Exploration:**7:                 **if** rand < ε8:                         Update the position of red-billed blue magpie by Equation (1)9:                 **else**10:                          Update the position of red-billed blue magpie by Equation (2)11:                 **end if**12:             **Exploitation:**13:                 **if** rand < ε14:                          Update the position of red-billed blue magpie by Equation (4)15:                 **Else**16:                          Update the position of red-billed blue magpie by Equation (5)17:                 **end if**18:            $\mathrm{Update}\;\mathrm{the}\;X^{\mathrm{food}}(t)$ and accomplish food storage by Equation (6)19:          **end while**20:          **return** best solution21:**end**

## 3. Improved Red-Billed Blue Magpie Optimization Algorithm

### 3.1. Circle Chaotic Mapping

The initial positions of the population are generated randomly in the RBMO algorithm, which cannot cover the entire search space effectively and results in suboptimal performance. The Circle chaotic mapping method is introduced for population initialization to address this limitation. Chaotic mapping exhibits excellent ergodicity and uniformity, ensuring that the population is more evenly distributed in the search space and achieves better optimization performance than random initialization. The mathematical expression of the Circle chaotic map is given in Equation (7):

(7) $$x_{i+1}=\mathrm{mod}\left(x_{i}+a-\left(\frac{k}{2\pi }\right)\sin\left(2\pi x_{i}\right),1\right)$$

where $x_{i}$ denotes the current value of the chaotic sequence at the i-th iteration. Parameter a is the modulation parameter, which affects the traversal speed and uniformity of the sequence. When a<0.1, the sequence is prone to local contraction, reducing the coverage ability of the search space; when a>0.3, the chaotic characteristics of the system weaken, which is not conducive to maintaining the randomness of the sequence. Research [18,19,20] indicates that a=0.2 can achieve a better balance between chaos and stability, so we adopted this value. Parameter k is the nonlinear strength coefficient, used to adjust the amplitude and nonlinearity of the chaotic sequence. A smaller k value will weaken the chaos of the system, while an excessively large k value may cause the sequence to enter a periodic zone or diverge. Based on the literature and the preliminary experimental results of this paper, k=0.5 can maintain a large chaotic domain and enhance the diversity of the initial solution.

**Figure 1:**
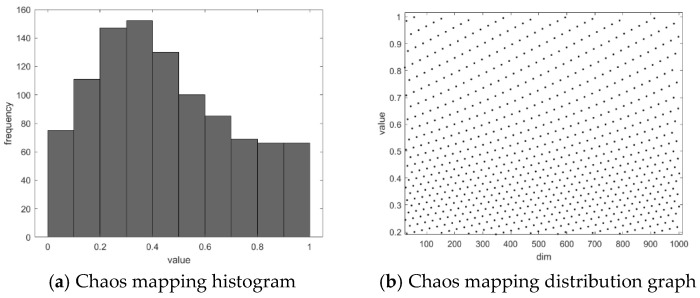
Circle chaotic mapping.

As shown in Figure 1, the left subfigure (a) represents the probability of each value, while the right subfigure (b) displays the chaotic points generated by the Circle chaotic mapping. It can be observed that using the Circle chaotic mapping to construct the initial population of the Red-billed Blue Magpie results in a more uniform distribution across the search space, higher population diversity, and an expanded search range for the algorithm.

### 3.2. Dynamic Adjustment of ε

The parameter ε serves as a key factor controlling whether individuals act in small groups or as a swarm when searching for food or attacking prey in the RBMO algorithm. The individuals tend to form small groups, focusing on local exploitation when the random number is less than ε. The individuals are more likely to act as a swarm, enhancing global exploration and helping the algorithm escape from local optima when the random number is greater than ε. The algorithm may suffer from insufficient exploration in the early stages and inadequate exploitation in the later stages if a fixed ε value is used. Therefore, dynamically adjusting ε helps avoid this issue by strengthening global search capability during the early phase and gradually converging toward local optimization in the later phase, thereby improving the overall solution quality of the algorithm.

**Figure 2:**
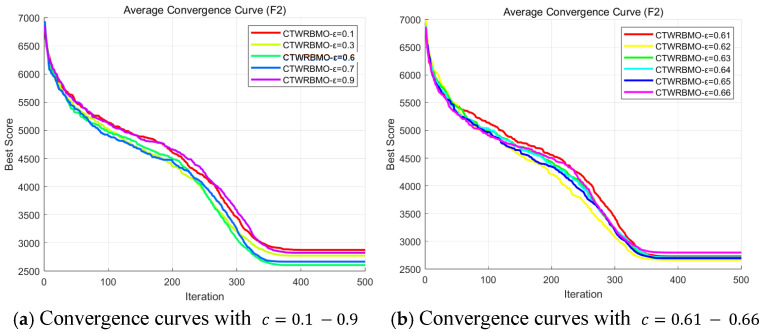
Average convergence curves for the sensitivity analysis of ε on CEC2020 F2.

The expression for ε is given in Equation (8).

(8) $$\varepsilon (t)=c\times \left(1-\left(\frac{t}{T}\right)\right)$$

where T represents the maximum number of iterations, and t denotes the current iteration number. Parameter c is the initial coefficient of the dynamic adjustment ε(t), which controls the global exploration intensity of the CTWRBMO algorithm in its early stage. To determine an appropriate initial value, we conducted a sensitivity analysis on parameter c using the CEC2020 benchmark function F2. For each candidate value, the algorithm was independently executed 30 times, and the convergence behavior was recorded. The results (Figure 2) show that c approximately equal to 0.62 provides the best balance between exploration and exploitation, yielding stable convergence and superior average performance., c = 0.62 is selected as the initial coefficient of ε(t) in CTWRBMO.

### 3.3. Nonlinear Dynamic Weight Factor wd

The disturbance amplitude during the position updating process significantly affects the search performance in the (RBMO) algorithm. The nonlinear dynamic weight factor wd, based on an exponential function, is introduced to achieve a more effective balance between global exploration and local exploitation. The value of wd is close to 1 during the early iterations, maintaining a strong disturbance intensity that encourages individuals to perform large-scale jumps within the solution space, which promotes global search and helps avoid local optima. The value of wd gradually decreases toward 0 in the later stages, limiting the disturbance amplitude to enhance local exploitation and accelerate convergence.

The expression of $w_{d}$ is given in Equation (9).

(9) $$\mathrm{wd}=\frac{e^{4\left(1-\frac{t}{T}\right)}-1}{e^{4\left(1-\frac{t}{T}\right)}+1}\;$$

where T represents the maximum number of iterations, and t denotes the current iteration number.

### 3.4. Elite Perturbation and Elite Retention Strategy Based on Individual Neighborhood

All individuals use the current global best solution Xfood as the guiding reference in the original (RBMO) algorithm, causing the entire population to converge toward it. Although this approach accelerates convergence, it easily leads the population to cluster in a single region and fall into local optima. Therefore, instead of directly using the global best position Xfood to guide the population, each individual generates a new locally perturbed candidate solution $X^{\mathrm{food}\_i}$ based on its neighborhood information. Then, following a greedy criterion, the algorithm determines whether to replace the current individual’s position. This mechanism prevents the population from converging excessively toward the best global position and improves search diversity and local exploitation capability.

During the foraging phase, Red-Billed Blue Magpies search for food either in groups or clusters. When cooperating in groups, each individual generates a new solution based on the positional differences of selected members, as defined in Equation (10):

(10) $$X^{\mathrm{food}\_i}\left(t+1\right)=X^{i}\left(t\right)+\left(\frac{1}{p}\times \sum_{m=1}^{p}X^{m}\left(t\right){-X}^{\mathrm{rs}}\left(t\right)\right)\times {\mathrm{Rand}}_{2}\times \mathrm{wd}$$

When the search is performed in a clustered form, the update rule is given as follows:

(11) $$X^{\mathrm{food}\_i}\left(t+1\right)=X^{i}\left(t\right)+\left(\frac{1}{q}\times \sum_{m=1}^{q}X^{m}\left(t\right)-X^{\mathrm{rs}}\left(t\right)\right)\times {\mathrm{Rand}}_{3}\times \mathrm{wd}$$

Both strategies generate perturbed solutions by constructing neighborhood-based difference vectors centered on the current individual $X^{i}$, rather than being guided by the global best solution Xfood.

After each perturbation update, the algorithm applies a greedy criterion to determine whether to accept the newly generated solution. If the fitness of $X^{\mathrm{food}\_i}$ is better than that of the original position $X^{i}$, the new position replaces the old one; otherwise, the previous solution is retained, as shown in Equation (12):

(12) $$X^{\mathrm{food}\_i}\left(t+1\right)=\left\{\begin{array}{c} X^{i}\left(t\right)\;\;if\;{\;\mathrm{fitness}}_{\mathrm{old}}^{i}>{\mathrm{fitness}}_{\mathrm{new}}^{i}\\ X^{food\_i}\left(t+1\right)\;\;\;\;\;else \end{array}\right.$$

During the prey-attacking phase, the same local perturbation mechanism is used to generate new solutions, and a coefficient CF is introduced to refine the perturbation of candidate solutions, as expressed in Equations (13) and (14):

(13) $$X^{\mathrm{food}\_i}\left(t+1\right)=X^{i}\left(t\right)+\mathrm{CF}\times \left(\frac{1}{p}\times \sum_{m=1}^{p}X^{m}\left(t\right)-X^{i}\left(t\right)\right)\times {\mathrm{Randn}}_{1}\times \mathrm{wd}$$

(14) $$X^{\mathrm{food}\_i}\left(t+1\right)=X^{i}\left(t\right)+\mathrm{CF}\times \left(\frac{1}{q}\times \sum_{m=1}^{q}X^{m}\left(t\right)-X^{i}\left(t\right)\right)\times {\mathrm{Randn}}_{2}\times \mathrm{wd}$$

All the above update operators are formulated in terms of three-dimensional position vectors $\left(x_{m},y_{m},z_{m}\right)$. The movement direction of each individual in the three-dimensional search space is determined by neighborhood difference vectors, while the displacement magnitude is jointly regulated by random perturbation terms and dynamic weighting factors. In this way, the search process can achieve a smooth transition from large-scale global exploration to refined local exploitation within a three-dimensional environment.

During the update process, a newly generated three-dimensional position is accepted only if it leads to an improvement in fitness; that is, a greedy selection strategy is adopted to ensure that the path solutions continuously converge toward better optima in the three-dimensional search space.

The algorithm applies a greedy criterion to determine whether to accept the newly generated solution after each perturbation update. In addition, the algorithm records the current best individual $X_{\mathrm{best}}$ in each iteration and outputs it as the final solution, ensuring the optimality and stability of the search results.

**Figure 3:**
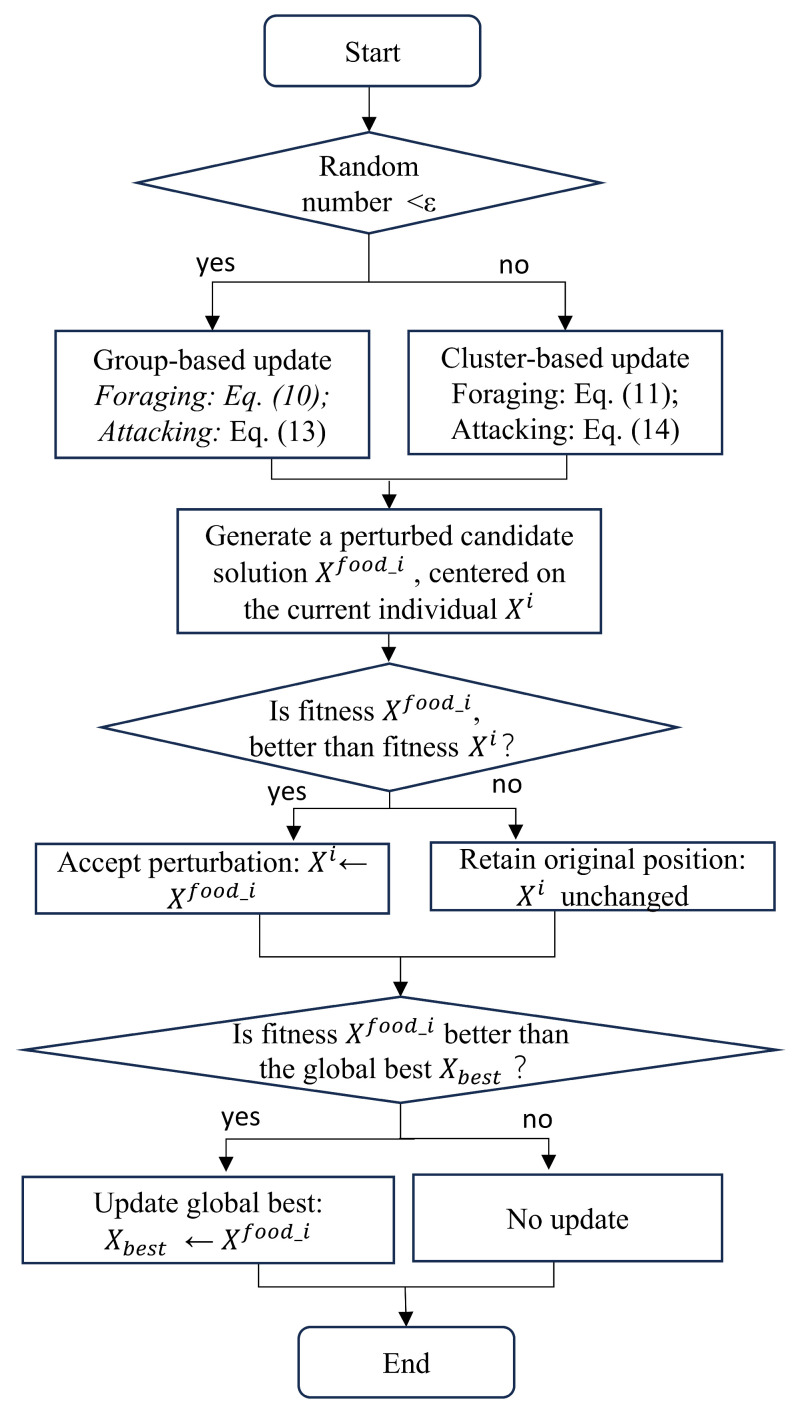
Flowchart of the elite disturbance mechanism.

To further improve the interpretability of the elite disturbance mechanism, the overall workflow of this mechanism is illustrated in Figure 3. The flowchart summarizes the individual neighborhood-based perturbation strategies corresponding to Equations (10), (11), (13) and (14), clearly showing that each candidate solution is generated by perturbing the individual position $X^{i}$, rather than being directly guided by the global best solution $X_{\mathrm{food}}$.

The following pseudocode (Algorithm 2) represents the detailed algorithmic procedure:
**Algorithm 2** The CTWRBMO1:**Begin**2:Initialize the relevant parameters (T, N, ε, wd, etc.) 3:Initialize population using Circle chaotic map (Equation (7))4:Evaluate initial fitness of all individuals5:$\mathrm{Set}\;\mathrm{global}\;\mathrm{best}\;X_{\mathrm{best}}$← best initial solution6:     **while** t < T7:Calculate the fitness of each search agent8:                $\mathrm{Update}\;\mathrm{global}\;\mathrm{best}\;\mathrm{solution}\;X_{\mathrm{best}}\;$ if improved9:                Dynamically update ε by (8)10:                Dynamically updated wd by (9)11:                **Exploration:**12:                     **if** rand < ε13:                             Generate candidate $X^{\mathrm{food}\_i}$ using Equation (10) 14:                     **Else**15:                             Generate candidate $X^{\mathrm{food}\_i}$ using Equation (11) 16:                     **end if**17:                **Exploitation:**18:                     **if** rand < ε19:                             Update red-billed blue magpie position using Equation (13)20:                     **Else**21:                             Update red-billed blue magpie position using Equation (14)22:                     **end if**23:                     Apply greedy selection by Equation (12)24:                     $\mathrm{Update}\;X_{\mathrm{best}}$ if new solution is better25:     **end while**26:     **Return global best solution**
$X_{\mathrm{best}}$
27:**end**

### 3.5. Comparison Between Original RBMO and CTWRBMO

To further highlight the improvement strategies proposed in this paper, a systematic comparison between the original RBMO and CTWRBMO is conducted at both the mathematical structure and algorithmic mechanism levels. As shown in Table 1, the differences between the two in key aspects such as initialization methods, step size adjustment, search updates, and elite retention are summarized.

The original RBMO employs uniform random initialization, which, although simple, results in substantial randomness in the population’s initial spatial coverage. This issue becomes more pronounced when the search space is large or the terrain is highly variable, making it difficult to ensure adequate initial solution quality. To address this limitation, CTWRBMO replaces random initialization with the Circle chaotic map, whose strong ergodicity and uniformity enable more thorough coverage of the search space during the initialization stage. This enhancement improves the initial population quality and reduces the sensitivity of convergence speed to initial distribution.

During the search phase, RBMO uses a fixed search probability ε, leading to an invariant balance between exploration and exploitation throughout the iteration process. While such a setting may yield stable performance on simple functions, it becomes inadequate in complex constrained environments, where a fixed ε cannot simultaneously support early-stage global exploration and late-stage local refinement. In contrast, CTWRBMO adopts a linearly decreasing ε(t), which starts at a relatively large value to strengthen global search in the early iterations and gradually decreases to facilitate fine-grained exploitation later on. This mechanism provides a more adaptive balance between exploration and exploitation.

The binary limitation of RBMO is also reflected in its step-size control. The disturbance amplitude changes only slightly during iterations, making it difficult for the algorithm to achieve both fast convergence and sufficient refinement accuracy. CTWRBMO introduces a nonlinear decaying weight factor, wd, to regulate the step size through exponential attenuation. This not only helps expand the search range in the early iterations but also enables finer adjustments near high-quality solutions as the search converges.

Furthermore, the position update in RBMO relies primarily on the global best individual. As iterations progress, such single-directional guidance tends to make the population converge prematurely, reducing diversity and increasing the risk of being trapped in local optima. To mitigate this, CTWRBMO incorporates neighborhood-based differential perturbation and employs a greedy selection mechanism. By integrating local neighborhood candidates while preserving elite solutions, CTWRBMO maintains necessary perturbations during the search, prevents premature convergence, and enhances search stability.

Overall, CTWRBMO introduces targeted improvements to initialization, exploration–exploitation regulation, and position update mechanisms, enabling it to achieve superior convergence stability and optimization accuracy in complex terrain environments.

### 3.6. Algorithm Time Complexity Analysis

Time complexity is an important indicator for evaluating algorithm performance. Let the problem dimension be D, and let the computational complexity of a single objective function evaluation be f(D). The initialization stage of CTWRBMO includes chaotic mapping and fitness evaluation, with a complexity of O(ND+Nf(D)). During the position update stage, each individual performs two update operations, each requiring the computation of the mean value over a subset of size q∈[10,N]. In the worst case, the update complexity is $O(N^{2}D)$. Meanwhile, each iteration involves 2N objective function evaluations, resulting in a complexity of O(Nf(D)).

The chaotic initialization, dynamic weighting factor, and exponential convergence factor introduce only constant-level or O(ND)-level computational overhead and therefore do not change the overall order of complexity. Consequently, the overall time complexity of CTWRBMO is $O(T(N^{2}D+Nf(D)))$, which is of the same order as that of the original RBMO algorithm.

## 4. Algorithm Performance Evaluation

### 4.1. Algorithm Parameter Settings

This study selects eight representative algorithms to comparatively evaluate the performance of the proposed CTWRBMO, including the classical benchmark algorithms PSO [21] and Grey Wolf Optimizer (GWO) [22]; recently developed advanced swarm intelligence optimization algorithms HHO [23], Whale Optimization Algorithm (WOA) [24], Gorilla Troops Optimizer (GTO) [25], and AVOA [26]; the state-of-the-art algorithm L-SHADE [27]; and RBMO as a baseline algorithm. These comparisons are conducted to validate the practical effectiveness of CTWRBMO and to assess the impact of the proposed improvements on algorithmic performance. The parameter settings of all algorithms are summarized in Table 2.

### 4.2. Performance Comparison of Algorithms

Systematic tests and assessments were conducted based on the CEC2020 benchmark function set (detailed in Table 3) to evaluate the performance of the improved RBMO algorithm, comparing CTWRBMO with multiple algorithms, including PSO, AVOA, WOA, GTO, GWO, L-SHADE, and RBMO. Each benchmark function was independently executed 30 times to rigorously examine the convergence characteristics and optimization capabilities of CTWRBMO, and the experimental data are presented in Table 4. To ensure fair comparison and reliability of the experimental results, all algorithms involved in the analysis were uniformly configured with a population size of N = 30, a maximum of T = 500 iterations, and a problem dimension of D = 20, minimizing the influence of the inherent stochasticity of heuristic algorithms on performance evaluation. The performance of each algorithm was comprehensively analyzed and compared using measures such as the best value, worst value, and mean value. All algorithms were implemented and simulated using MATLAB R2022a on a platform equipped with an AMD Ryzen 7 8845H processor (Advanced Micro Devices, Inc., Santa Clara, CA, USA), with a base clock speed of 3.80 GHz. 

The CEC2020 benchmark suite consists of 10 single-objective test functions, categorized into unimodal functions (F1), basic functions (F2–F4), composition functions (F5–F7), and hybrid functions (F8–F10), where $F_{i}^{*}$ denotes the optimal value of the i-th test function.

The experimental results of different algorithms on the CEC2020 benchmark functions are presented in Table 4. Overall, the proposed CTWRBMO algorithm demonstrates superior convergence performance and stronger robustness on most test functions. Specifically, on functions F1, F4, and F6, the optimal values obtained by CTWRBMO are very close to the theoretical optima; meanwhile, on functions F3, F5, F7, F8, F9, and F10, its best solutions consistently outperform those of all comparison algorithms, indicating that the proposed algorithm achieves higher convergence accuracy on these problems.

The mean values of the algorithms reflect their overall optimization performance over 30 independent runs. As shown in Table 4, CTWRBMO achieves the best average performance on F1, F2, F3, F4, F7, F8, and F9, demonstrating its ability to stably obtain high-quality solutions across repeated experiments. In addition, for most test functions, CTWRBMO yields significantly smaller standard deviations than the other algorithms, indicating lower performance variability and stronger robustness. These statistical results suggest that CTWRBMO is able to maintain stable convergence behavior and reliable solution quality across different runs.

**Figure 4:**
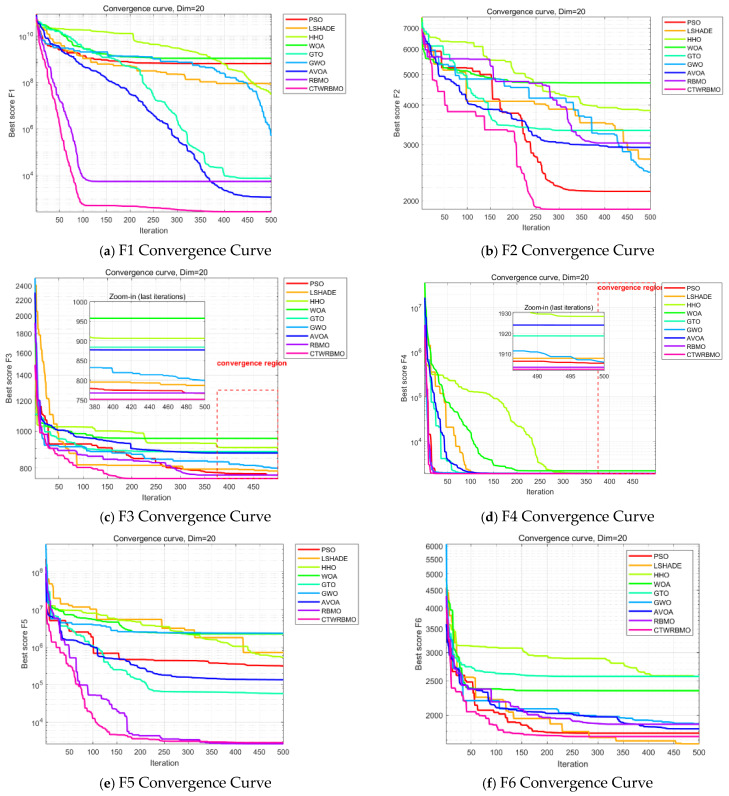

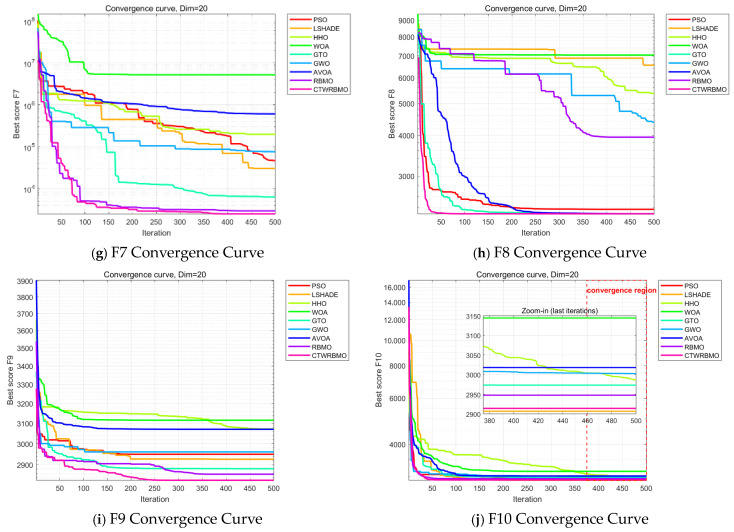
Convergence curves of seven algorithms on CEC2020 test functions.

Figure 4 presents the average convergence curves of the nine algorithms on the test functions. These results are based on the mean performance obtained from 30 independent runs of each algorithm and provide an intuitive comparison of convergence speed and accuracy. As can be observed, CTWRBMO exhibits a more pronounced downward trend in the early iterations, indicating a faster convergence speed at the initial stage. On F1, F2, F3, F4, F7, F8, and F9, the convergence curves of CTWRBMO consistently lie below those of all comparison algorithms, demonstrating higher final solution accuracy.

It should be noted that on the test functions F6 and F10, the final convergence accuracy of CTWRBMO is slightly inferior to that of L-SHADE, and on F5, it is slightly inferior to RBMO, indicating that its performance advantage is not absolute on all test functions. However, the differences in the final optimal values among the algorithms on these functions are relatively small, and CTWRBMO still maintains a faster overall convergence speed, suggesting that it remains highly competitive for complex optimization problems.

Overall, CTWRBMO demonstrates faster convergence, higher solution accuracy, and better stability on most test functions, while its performance advantage is relatively weakened on a few functions, indicating that the algorithm still exhibits certain performance variations under different problem characteristics.

### 4.3. Wilcoxon Rank-Sum Test

In this study, the Wilcoxon rank-sum test was employed to statistically analyze the best results obtained from 30 independent runs, with the aim of evaluating the performance differences between the proposed CTWRBMO algorithm and the comparative algorithms. A significance level of *p* < 0.05 was adopted to indicate statistically significant differences between two algorithms. The statistical results are summarized in Table 5. For most benchmark functions from F1 to F10, the *p*-values between CTWRBMO and the other algorithms are lower than 0.05, demonstrating that the performance improvement of CTWRBMO is statistically significant. These results indicate that CTWRBMO exhibits significant advantages in terms of optimization accuracy and stability. Overall, CTWRBMO significantly outperforms the other algorithms on the majority of the test functions, and the observed performance improvements are supported by clear statistical evidence, thereby further validating the effectiveness and robustness of the proposed method.

### 4.4. Ablation Experiments

To verify the effectiveness of the proposed strategies, ablation experiments were conducted in this study. Four different algorithm variants were constructed by incorporating each strategy individually into the RBMO framework. Specifically, IRBMO1 incorporates only the Circle chaotic mapping, IRBMO2 incorporates only the nonlinear dynamic weighting factor wd, IRBMO3 incorporates only the ε parameter, and IRBMO4 incorporates only the elite perturbation and elite retention strategy based on individual neighborhoods.

Six representative benchmark functions from CEC2020 (F2, F3, F4, F7, F9, and F10) were selected for performance evaluation. In all experimental settings, algorithmic parameters were strictly kept identical. Each algorithm was independently executed 30 times, with a maximum of 500 iterations per run, in order to reduce the influence of randomness. To intuitively compare the contribution of each strategy, the symbols “+/=/−” were used to summarize the relative performance of each algorithm compared with the original RBMO in terms of average optimization results. Specifically, “+” indicates the number of cases in which the algorithm outperforms RBMO, “=” denotes equivalent performance, and “−” indicates inferior performance relative to RBMO.

As shown in Table 6, the “+/=/−” statistics of IRBMO1 are 17/1/0, indicating that IRBMO1 achieves superior average convergence accuracy compared with RBMO on most benchmark test functions. Since RBMO itself can reach the theoretical optimum on function F4, IRBMO1 also converges to this theoretical optimum, resulting in one “=”. This result demonstrates that circular chaotic initialization can effectively enhance the initial quality of the population, allowing the search process to start from more promising initial positions and thereby improving the overall convergence performance of the algorithm.

**Figure 5:**
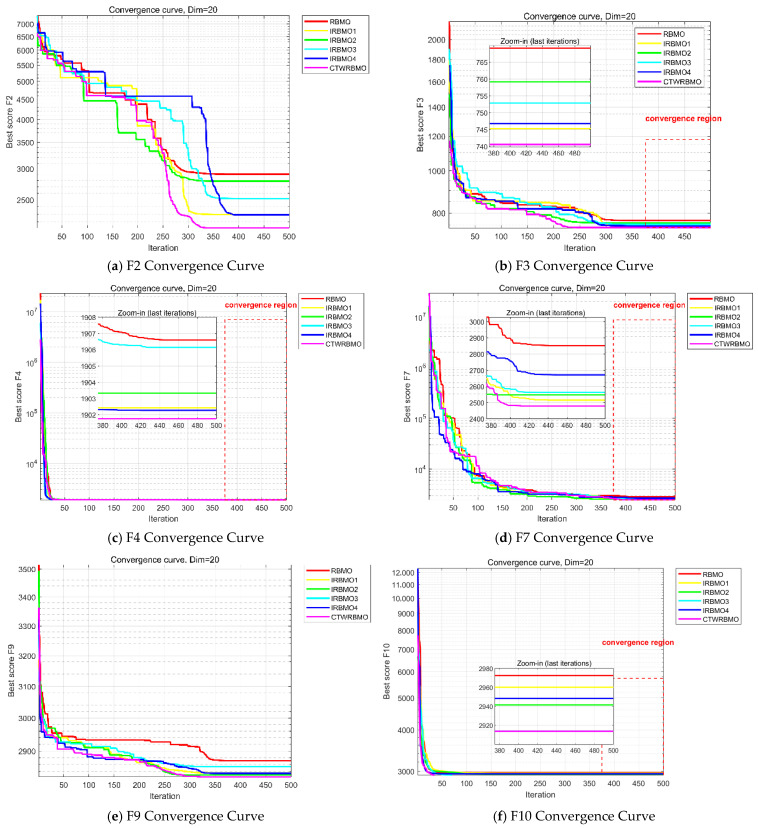
Convergence curves of the ablation experiment.

The “+/=/−” value of IRBMO2 is likewise 17/1/0, and it can be observed from Figure 5 that IRBMO2 converges significantly faster than RBMO. This is because, during the early stages of iteration, the nonlinear weighting factor maintains a relatively large value, which enlarges the search step size and enables the population to rapidly move toward regions with high-quality solutions, thereby markedly accelerating the overall convergence speed.

The “+/=/−” statistics of IRBMO3 are also 17/1/0. Although IRBMO3 exhibits faster convergence on some functions (e.g., F7 and F9) and slightly slower convergence than RBMO on the remaining functions, it achieves higher final average convergence accuracy, with standard deviations consistently lower than those of RBMO. This indicates that the dynamic ε strategy can effectively enhance both the stability of the algorithm and the quality of the obtained solutions, and thus has considerable value as an improvement strategy.

The “+/=/−” value of IRBMO4 is 17/1/0, and its convergence accuracy is likewise superior to that of RBMO. As observed in Figure 5, IRBMO4 is able to escape from local optima multiple times. This is mainly attributed to the introduction of the elite disturbance mechanism, which generates perturbed candidate solutions based on the current individual position $X^{i}$ and its neighborhood information, and then determines whether to accept the new solutions according to a greedy criterion. This mechanism prevents the population from prematurely concentrating in a single optimal region while simultaneously enhancing local search accuracy, thereby improving population diversity and the ability to escape local optima when solving complex optimization functions.

## 5. Problem Formulation

### 5.1. Problem Description

UAV path planning aims to find an optimal flight trajectory from the start point to the target point while satisfying the UAV’s operational constraints, such as maximum and minimum flight distance, turning angles, and threat zones. The planned path must not only account for external environmental factors, including terrain variations and threat areas, but also adhere to the UAV’s fundamental performance limitations, encompassing maximum range, minimum segment length, maximum turning angle, and allowable speed range.

### 5.2. 3D UAV Flight Environment Modeling

**Figure 6:**
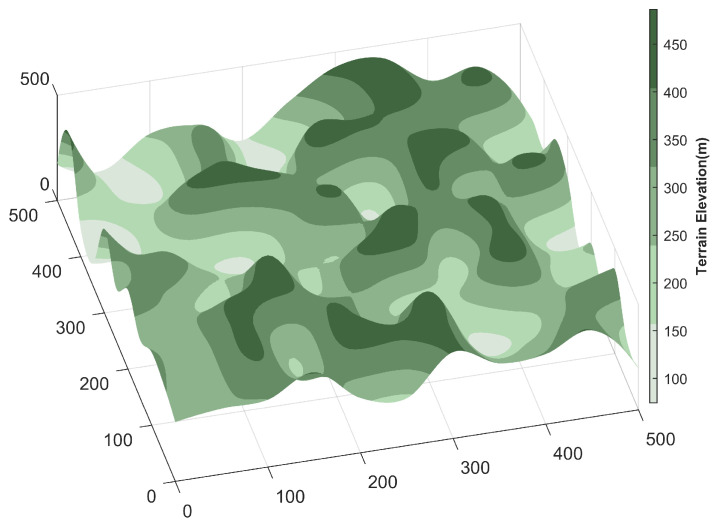
The 3D terrain map.

Before executing a mission, the UAV must first model the environment to simulate its actual flight conditions. In constructing the environment model, this study assumes static terrain, fixed obstacles, and no wind interference, thereby considering only the UAV’s flight characteristics in a static environment. The integrated smoothing algorithm proposed in [28] satisfies the UAV’s longitudinal maneuverability constraints while closely fitting the original terrain and slightly elevating it, resulting in a safer and more reliable path planning process. In this study, the integrated smoothing algorithm was employed to model the terrain, resulting in an equivalent terrain surface Z(x, y). To ensure that the UAV maintains the necessary vertical safety clearance throughout the flight, a minimum safe altitude h above the equivalent terrain is added to account for potential collision risks due to terrain variations. This yields a modified terrain surface H(x, y), as illustrated in Figure 6, which is expressed as follows:

(15) H(x, y) = Z(x, y) + h

### 5.3. Constraint Conditions

#### 5.3.1. Maximum Flight Distance Constraint

UAVs are subject to range constraints during flight due to limited energy resources (battery or fuel), and the flight path must respect the maximum allowable distance. The maximum safe flight distance is assumed to be $L_{\max}$ to ensure mission completion and avoid energy depletion.

The total flight distance must satisfy the following constraints:

(16) $$\sum_{i=0}^{node}l_{i}\leq L_{max}$$

Here, node denotes the number of flight segments, and $l_{i}$ represents the flight distance of the i-th segment.

#### 5.3.2. Minimum Flight Distance Constraint

The flight distance $l_{i}$, between any two consecutive flight segments is not excessively short to avoid frequent directional changes during flight, which increase energy consumption, the drone must ensure that. Assuming a minimum flight distance of $L_{\min}$, each segment’s distance must satisfy:

(17) $$l_{i}\geq L_{min}$$

#### 5.3.3. Maximum Turning Angle Constraint

At each path waypoint, a UAV must adjust its heading based on the directional change between the preceding and succeeding flight segments. If the turning angle is too large, the UAV may be unable to generate sufficient centripetal force through attitude adjustment to complete the maneuver, potentially leading to attitude oscillations, path deviations, or even stall. Therefore, it is necessary to set an upper limit, $\Psi_{max}$, on the maximum turning angle between consecutive flight segments to ensure trajectory feasibility and flight stability. For a sequence of path waypoints $n_{1},n_{2},\ldots ,n_{N}$, the flight direction vectors before and after waypoint $n_{r}$ are given by $\left(n_{r}-n_{r-1}\right)$ and $\left(n_{r+1}-n_{r}\right)$, respectively, and the UAV’s turning angle at waypoint $n_{r}$, $\Psi_{r}$, is calculated as the angle between these two vectors:

(18) $$Cos\Psi_{r}=\frac{\left(n_{r}-n_{r-1}\right)\cdot \left(n_{r+1}-n_{r}\right)}{\left\|n_{r}-n_{r-1}\right\|\left\|n_{r+1}-n_{r}\right\|}$$

To avoid sharp turns and ensure a feasible flight, the turning angle must satisfy the following constraint:

(19) $$\Psi_{r}\leq \Psi_{max}$$

Here, $\Psi_{\max}$ denotes the maximum safe turning angle that the UAV can achieve while remaining within the allowable limits of its attitude, lateral acceleration, and structural load-bearing capacity.

#### 5.3.4. Maximum Climb/Descent Rate Constraints

When the altitude difference between consecutive path nodes is too large, the UAV may not be able to adjust its height within the available time, leading to safety risks such as insufficient thrust due to excessive climbing or aerodynamic instability caused by rapid descent. Therefore, it is necessary to impose a maximum climb rate $v_{climb,max}$ and a maximum descent rate $v_{descent,max}$ during path planning to ensure that the trajectory remains vertically feasible. Let the altitudes of consecutive path nodes be $z_{r}$ and $z_{r+1}$, and let the flight time interval be Δt; the vertical velocity along this segment is given by

(20) $$v_{z,r}=\frac{z_{r+1}-z_{r}}{\Delta t}$$

The vertical velocity of the UAV is required to satisfy the following constraint:

(21) $$-v_{descent,max}\leq v_{z,r}\leq \;v_{climb,max}$$

where $v_{z,r}$ denotes the UAV’s average vertical velocity along the r-th flight segment, ensuring that the altitude change remains within a controllable range.

#### 5.3.5. Flight Speed Constraints

The flight speed of a UAV is generally influenced by factors such as the propulsion system thrust, aerodynamic drag, endurance, and structural limitations of the aircraft. Flying at too low a speed may pose a risk of stall, while excessively high speeds may result in structural overload or unstable maneuvering. Therefore, it is necessary to impose speed limits for each segment in the planned path.

Let the spatial distance between two consecutive nodes $n_{r}$ and $n_{r+1}$ be $∥n_{r+1}-n_{r}∥$, and Δt denote the assumed time interval between these nodes. The average speed along this segment can then be expressed as follows:

(22) $$v_{r}=\frac{\left\|n_{r+1}-n_{r}\right\|}{\Delta t}$$

The flight speed should satisfy the following constraint:

(23) $$v_{min}\leq v_{r}\leq v_{max}$$

where $v_{min}$ and $v_{max}$ represent the allowable minimum and maximum safe flight speeds, respectively, ensuring that the UAV maintains controllability and stability throughout the entire flight path.

#### 5.3.6. Collision Avoidance Constraint

**Figure 7:**
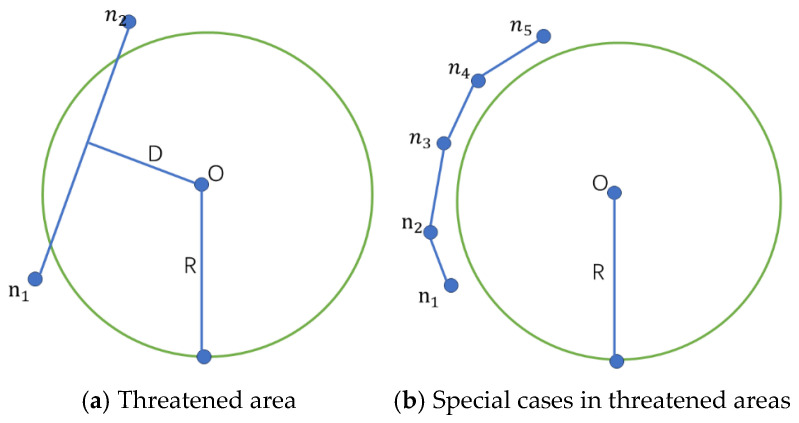
Threat model.

Unmanned aerial vehicles (UAVs) may encounter various obstacles such as mountains, hills, and no-fly zones during path planning. This paper employs spherical objects to simulate obstacles, as illustrated in Figure 7a. Let a threat area be centered at O with radius R; the interior of this sphere is strictly prohibited for UAV entry. Suppose a UAV is expected to travel from node $n_{1}$ to node $n_{2}$. Although $n_{1}$ and $n_{2}$ themselves are located outside the threat region, the flight path between them may intersect the spherical threat area centered at O with radius R (see Figure 7b). To prevent the UAV from intruding into this restricted zone, collision avoidance measures must be applied. In this study, additional path nodes are dynamically inserted between $n_{1}$ and $n_{2}$ to enable the UAV to smoothly bypass the threat region and avoid potential hazards.

To achieve this, the path segments are refined using a uniform node distribution approach. Let the straight-line distance from the start point to the end point be L, and let the desired spacing between adjacent path nodes be l. The number of additional nodes required along the path can then be calculated as

(24) $$n=\frac{L}{l}$$

By refining the path nodes, the UAV can perform more precise collision detection with threat areas during the path optimization process, ensuring that the generated flight path does not enter spherical threat zones and thereby satisfying the collision avoidance constraints.

### 5.4. Cost Function

In the process of unmanned aerial vehicle (UAV) path planning, the cost function serves as the criterion for evaluating the relative merits of different paths. A well-designed cost function guides the algorithm to generate safe and efficient paths within complex environments, avoiding terrain obstacles and hazardous zones. This paper constructs the following UAV path cost function by comprehensively considering multiple factors, including maximum flight range, minimum flight altitude, turn angle, and avoidance of hazardous zones:

(25) $$F_{cost}={\left(F_{dist}\right)}^{\omega_{1}}\times {\left(F_{high}\right)}^{\omega_{2}}{\times (F_{angle})}^{\omega_{3}}\times {\left(F_{threat}\right)}^{\omega_{4}}$$

The coefficients ω_1_, ω_2_, ω_3_, and ω_4_ represent the weights assigned to distance, altitude, turning angle, and threat costs, respectively. Following the principles of safety and operational efficiency, these weight coefficients are set as follows:

Threat weight ω_4_ = 5: The threat cost is the most critical safety constraint in path planning. Entering a threat zone may result in mission failure; therefore, the threat cost is assigned the highest weight to ensure that the algorithm always prioritizes path safety.

Distance weight ω_1_ = 1: Path length directly affects UAV energy consumption and mission duration. As a key factor secondary only to safety, it is assigned a moderately high weight to encourage the algorithm to search for shorter paths.

Altitude weight ω_3_ = 0.5: The turning angle cost mainly influences path smoothness and flight maneuverability. Its importance is lower than that of threat avoidance and path length, so it is assigned a moderate weight to prevent unnecessary sharp turns while avoiding excessive penalization of path curvature.

Angle weight ω_2_ = 0.05: The magnitude of the altitude cost is typically much higher than other cost terms. Without proper normalization, it could dominate the optimization process. Therefore, a relatively small weight is assigned to ensure safe altitude while minimizing its interference with distance and threat optimization.

#### 5.4.1. Distance Cost

The path distance directly affects the UAV’s energy consumption and flight time; therefore, it is essential to consider the distance between each consecutive path node during path planning. The distance cost term, $F_{dist}$, can be obtained by summing the Euclidean distances of all consecutive segments along the path, which is calculated as follows:

(26) $$F_{dist}=\left\|\left.n_{r}-n_{r-1}\right\|\right.+\left\|\left.n_{r+1}-n_{r}\right\|\right.$$

Here, $∥n_{r}-n_{r-1}∥$ represents the spatial distance between two consecutive nodes $n_{r}$ and $n_{r-1}$, while $∥n_{r+1}-n_{r}∥$ denotes the spatial distance between the consecutive nodes $n_{r}$ and $n_{r+1}$.

#### 5.4.2. Angular Cost

In UAV path planning, the smoothness of path turning plays a critical role in ensuring flight stability and reducing maneuvering load. Excessively large turning angles can not only increase lateral overload but also lead to abrupt maneuvers, higher energy consumption, and in some cases, infeasible flight actions. Therefore, in this study, an angular cost term, $F_{\mathrm{angle}}$, is introduced into the objective function to penalize large turning angles along the path, thereby improving the overall path smoothness. The angular cost term is calculated as follows:

(27) $$F_{angle}=\left\{\begin{array}{c} 50,\;\;\;\;\;\;\;\;\;\;\;\;\;\;\;\;\;\;\;\;\;\;\;\;\;\;\;\;\;\;\;\;\;\;\;\;\;\;\;\;\;\mathrm{Cos}\Psi_{r}\geq 0\\ 10\left(2+Cos\Psi_{r}\right),\;\;\;\;\;\;\;\;\;\;\;\;\;\;\;\mathrm{Cos}\Psi_{r}<0 \end{array}\right.$$

#### 5.4.3. Altitude Cost

During the UAV’s flight from the start point to the endpoint, both terrain clearance and altitude constraints must be satisfied. To ensure flight safety, the UAV should neither fly too low, which would increase the risk of collision, nor fly excessively high, which would result in unnecessary energy consumption. To address this, an altitude cost term is incorporated into the objective function to penalize unsafe altitude behaviors. If the flight path maintains a safe altitude and changes smoothly, the corresponding cost remains low. Conversely, if the flight altitude falls below the minimum allowable height h or approaches the terrain height $T(x_{r},y_{r})$, the cost increases significantly to avoid potential collision risks. Terrain elevation data are obtained from a pre-established three-dimensional digital elevation model (DEM). For any given path point $\left(x_{r},y_{r}\right)$, the corresponding terrain height $T(x_{r},y_{r})$ is determined through terrain interpolation and contributes to the computation of the altitude cost $F_{\mathrm{high}}$, as expressed in Equation (28).

(28) $$F_{high}=\left\{\begin{array}{c} z_{r}+P_{h}+P_{t},\;\;\;\;z_{r}<h\;\;\;\mathrm{and}\;\;z_{r}\leq T\left(x_{r},y_{r}\right)+\delta \\ \;\;z_{r}+P_{h},\;\;\;\;\;\;\;\;\;\;z_{r}\leq h\;\mathrm{and}\;\;z_{r}>T\left(x_{r},y_{r}\right)+\delta \\ \;\;z_{r}+P_{t},\;\;\;\;\;\;z_{r}>h\;\;\;\mathrm{and}\;\;z_{r}\geq T\left(x_{r},y_{r}\right)+\delta \\ \;z_{r},\;\;\;\;\;\;\;\;\;\;\;\;\;\;\;\;\;\;\;\;\;\;\;\;\;\;\;\;\;\;\;\;\;\;\;\;\;\;\;\;\;\;\;\;\;\;\;\mathrm{otherwise} \end{array}\right.$$

Here, $z_{r}$ denotes the flight altitude at the r-th path point, h represents the minimum allowable flight altitude, and $T(x_{r},y_{r})$ indicates the terrain elevation at the r-th point. The parameter δ defines the minimum safety distance to avoid collision with the terrain. $P_{h}=10^{3}$ is the penalty coefficient applied when the flight altitude falls below the minimum allowable altitude h, while $P_{t}=10^{5}$ is the penalty coefficient applied when the flight altitude is lower than the sum of the terrain height and the safety distance. These penalties are used to strictly prevent the UAV from flying through the terrain.

#### 5.4.4. Threat Costs

In practical flight environments, UAVs must strictly avoid obstacles, terrain, and no-fly zones. Therefore, this study incorporates a threat evaluation function $f_{\left(r,i\right)}$ into the cost function to determine whether any point $n_{r}$ along the path approaches or enters the i-th threat region.

(29) $$f_{r,i}=\left\{\begin{array}{c} 0,\;\;\;\;\;\;\left\|n_{r}-p_{i}\right\|<r_{i}+\delta_{s}\\ 1,\;\;\;\;\;\;\left\|n_{r}-p_{i}\right\|>r_{i}+\delta_{s} \end{array}\right.$$

Each threat source is modeled as a spherical region, where $p_{i}$ represents the center of the threat and $r_{i}$ denotes its radius. An additional safety buffer of $\delta_{s}=20\;m$ is applied around each sphere to mitigate the risk of boundary crossing due to positioning errors or airflow disturbances. When $f_{\left(r,i\right)}=0$, it indicates that the path intersects with an obstacle or a no-fly zone, and the path is immediately deemed infeasible. In this study, all threat sources are treated as forbidden regions; if any of these regions are violated, a penalty is imposed on the total cost function, as defined in Equation (30):

(30) $$F_{threat}=\left\{\begin{array}{c} 1+10\left(d-\sum_{i=1}^{d}f_{r,i}\right),\;\;\;\;\;\;\;\;\;\;\;\;\;\sum_{i=1}^{d}f_{r,i}<d\\ 1+0.1\times d,\;\;\;\;\;\;\;\;\;\;\;\;\;\;\;\;\;\;\;\;\;\;\sum_{i=1}^{d}f_{r,i}=d \end{array}\right.$$

Here, d denotes the total number of threat sources. If $\sum_{i=1}^{d}f_{r,i}<d$, it indicates that the path has entered an obstacle or a no-fly zone at least once, and consequently, $F_{\mathrm{threat}}$ is forcibly increased by approximately tenfold. Conversely, if $\sum_{i=1}^{d}f_{r,i}=d$, all path nodes are within safe regions, and a smaller penalty is applied to encourage maintaining a safe distance.

### 5.5. UAV 3D Path Planning Simulation and Analysis

**Figure 8:**
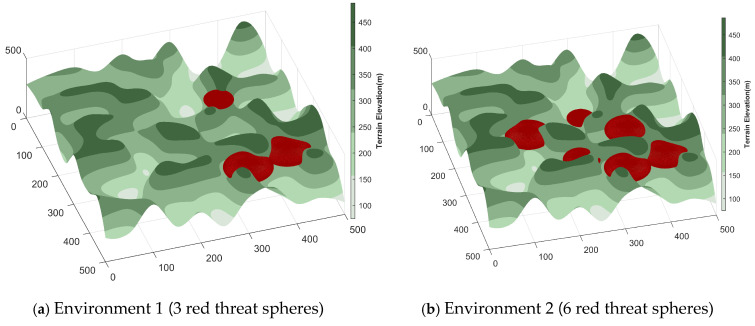
Three-dimensional terrain map (including threats).

In this work, three-dimensional UAV path planning experiments were performed using MATLAB R2022a. To enable the UAV to effectively circumvent terrain obstacles and reach the destination safely, the planning framework was developed on a 3D terrain model incorporating predefined threat regions. Two mountainous environments were constructed, containing three and six threat zones, respectively, to emulate flight scenarios of differing complexity and evaluate the CTWRBMO algorithm’s performance under various environmental conditions. The UAV’s operational domain was specified as a 500 × 500 × 500 spatial grid. The initial position was set to (15.15, 30.3, 295.9), while the destination point was defined as (449.5, 459.6, 422). A summary of the environmental configurations is presented in Table 7, and detailed parameters of the threat models are listed in Table 8. The corresponding 3D terrain model generated from these parameters is depicted in Figure 8.

In this study, three-dimensional UAV path planning in Environments 1 and 2 was investigated using five optimization algorithms—ABC, AVOA, HHO, RBMO, and the proposed CTWRBMO. The selection of these algorithms is based mainly on two considerations. First, ABC is a classical benchmark algorithm that provides a reliable baseline for comparison; HHO and AVOA are recently proposed algorithms that have demonstrated competitive performance in recent years; and RBMO is adopted as the baseline algorithm for improvement to verify the effectiveness of the proposed strategies. In addition, during preliminary exploratory experiments, we observed that in strongly constrained three-dimensional environments involving complex terrain, obstacles, and multiple kinematic constraints, general-purpose swarm intelligence algorithms such as PSO, GTO, and WOA have difficulty in stably generating feasible and safe paths when no additional constraint-handling or path-repair mechanisms are incorporated. To avoid compromising the fairness and consistency of the comparative study due to the use of different constraint-handling strategies across algorithms, these algorithms were not included in the three-dimensional path planning comparisons.

Furthermore, in order to enhance the ability of the algorithm to generate feasible solutions in a strongly constrained three-dimensional environment and to further improve the comparability among different algorithms, this study introduced the same initial path generation strategy for all the compared algorithms to construct feasible initial solutions. This initial path serves only as a starting reference for the search process and does not participate in the fitness evaluation during the subsequent path optimization process, thereby avoiding any bias impact on the final optimization result.

To further guarantee the comparability of the experimental results, the population size of all algorithms was set to 50, and the maximum number of iterations was fixed at 100. Each algorithm was independently executed 30 times in both Environment 1 and Environment 2. The optimal, mean, and variance of the path lengths obtained from these 30 independent runs are reported. A smaller mean indicates a lower average path planning cost, while the variance reflects the stability and robustness of the algorithms in three-dimensional environments.

In addition, multiple performance metrics were evaluated, including average computational time, path feasibility rate, path smoothness, and energy consumption, to comprehensively assess the performance and applicability of each algorithm. The energy consumption of each UAV path was calculated using a simplified model that considers the Euclidean distance between consecutive waypoints, the path curvature (which indirectly reflects the UAV’s maximum turning angle constraint), and vertical energy expenditure (which indirectly reflects climb and descent rates). Although simplified, this model provides a consistent and fair basis for comparing different algorithms. The parameter settings for all algorithms are summarized in Table 9.

**Figure 9:**
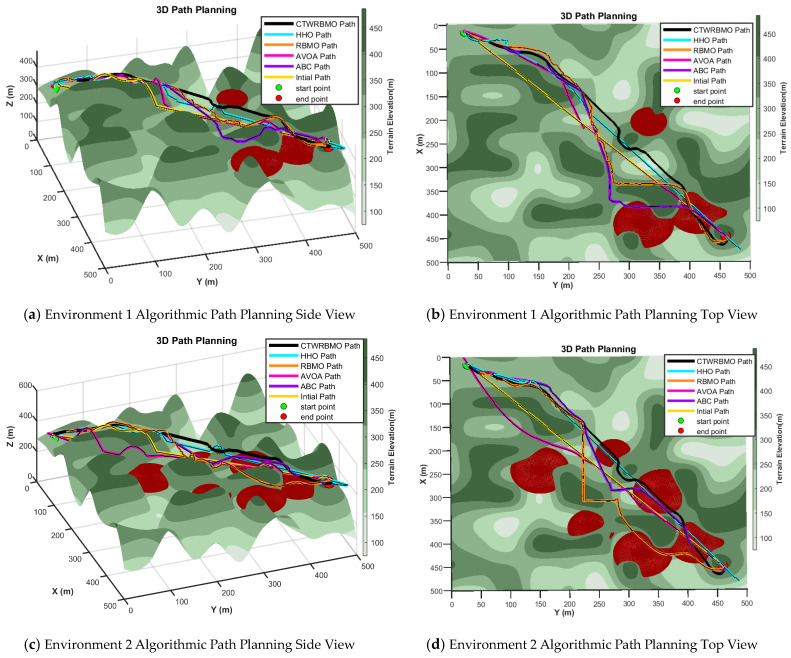
Path planning by different algorithms in various environments.

This study presents representative three-dimensional side views and top views of typical experimental results to facilitate an intuitive comparison of the performance of different algorithms in three-dimensional UAV path planning. The results for Environment 1 are illustrated in Figure 9a,b. In this scenario, the number of threat regions is relatively small. The path planned by the CTWRBMO algorithm exhibits overall altitude stability, with a smooth flight trajectory throughout the mission and no abrupt turning angles, demonstrating strong obstacle avoidance capability. In contrast, the UAV paths generated by the other algorithms show larger altitude fluctuations and tend to fly closer to mountainous terrain, indicating weaker obstacle avoidance and threat avoidance capabilities, which makes flight safety difficult to guarantee.

The results for Environment 2 are shown in Figure 9c, where a larger number of threat regions are present. Except for the CTWRBMO algorithm, the paths generated by the other algorithms exhibit significant altitude fluctuations and poor trajectory smoothness. Nevertheless, even in this more complex environment, the path produced by CTWRBMO maintains minimal altitude variation and superior smoothness, as further illustrated by the top view in Figure 9d. The paths generated by RBMO and ABC contain large turning angles, which increase the overall path length and result in sharper maneuvers. By comparison, the CTWRBMO algorithm produces a smoother and shorter path.

Table 10 summarizes the path planning performance of the different algorithms. In Environment 1, the average path cost of CTWRBMO is lower than that of RBMO, HHO, ABC, and AVOA by 6.04%, 36.23%, 4.29%, and 31.43%, respectively. In Environment 2, the reductions are 6.29%, 30.22%, 6.18%, and 31.38%, respectively, indicating that CTWRBMO is highly effective in minimizing path length.

Path success rate was used to evaluate the reliability of an algorithm in generating feasible paths. The results show that both CTWRBMO and ABC achieve a 100% success rate in both environments, meaning that all paths generated during 30 independent runs were feasible and did not pass through threat zones or produce abnormal trajectories. RBMO maintains a 100% success rate in Environment 1 but drops to 93% in Environment 2, suggesting reduced robustness under higher threat density. HHO and AVOA exhibit lower success rates, often generating paths that violate no-fly zones or contain excessive sharp turns.

Regarding path smoothness, CTWRBMO produces the smallest average turning angles, indicating that its trajectories are smoother compared to those generated by the other algorithms.

Energy consumption and average computational time were also evaluated. CTWRBMO consistently achieves the lowest energy consumption due to its shorter and smoother paths, which minimize frequent sharp-turn operations. In contrast, the other algorithms consume more energy because their paths are longer and less smooth, requiring more maneuvers. The average computational time of CTWRBMO is comparable to RBMO and slightly higher than ABC or AVOA, demonstrating that the improved solution quality does not incur a prohibitive computational cost.

It is noteworthy that CTWRBMO exhibits the smallest variation in path cost across independent runs, indicating minimal performance fluctuation and high stability. Overall, these results demonstrate that CTWRBMO can reliably generate high-quality, feasible, smooth, and energy-efficient paths while maintaining acceptable computational times, making it particularly suitable for complex 3D UAV path planning scenarios.

**Figure 10:**
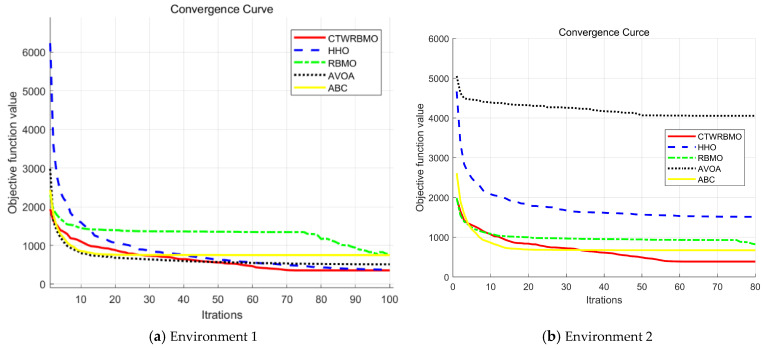
Iterative convergence curves of various algorithms under different conditions.

From the convergence curves of the different algorithms in Environment 1 (Figure 10a), it can be observed that during the first 25 iterations, CTWRBMO ranks third in convergence speed, slightly behind AVOA and ABC. However, as the iterations progress, by the 60th iteration, the convergence accuracy of CTWRBMO surpasses that of both AVOA and ABC, achieving the best overall performance among all algorithms. This indicates that, although CTWRBMO explores the solution space slightly more slowly in the early stage, its optimization strategies enable efficient solution refinement in subsequent iterations, ultimately achieving higher convergence accuracy.

Similarly, in Environment 2 (Figure 10b), CTWRBMO exhibits relatively slower convergence in the early iterations. Nevertheless, around the 45th iteration, its convergence accuracy exceeds that of ABC, rising to the top rank. These results demonstrate that CTWRBMO can effectively generate high-quality solutions in the later stages of iteration, highlighting its robustness and excellent global search capability across different 3D UAV path planning scenarios.

## 6. Conclusions

This paper presents an enhanced version of the Red-billed Blue-jay Optimization Algorithm (CTWRBMO), which integrates multiple strategies to improve the accuracy and computational efficiency of three-dimensional path planning. The introduction of Circle chaotic mapping during the population initialization phase enhances the diversity of initial solutions, effectively mitigating the risk of being trapped in local optima. In addition, the algorithm incorporates a dynamic ε adjustment mechanism and a nonlinear dynamic weight factor to balance global search and regional exploration, enabling extensive exploration and comprehensive exploitation of high-quality solutions. Furthermore, a local search strategy based on elite perturbation is proposed to enhance the stability and overall balance of the search process, effectively addressing the premature convergence issues commonly observed in the traditional RBMO algorithm.

Simulation results demonstrate that CTWRBMO consistently achieves the lowest average path cost across different environments with varying numbers of threat targets. The algorithm also exhibits the smallest variance in path cost, indicating high adaptability and stability in response to changes in initial conditions and environmental uncertainties. In terms of convergence speed and solution accuracy, CTWRBMO performs excellently, generating smooth, safe, and reliable optimal paths in complex and dynamic flight scenarios, as evidenced by convergence curve analyses.

The performance of CTWRBMO carries important practical implications for real UAV systems. By generating shorter and smoother paths, the algorithm effectively reduces energy consumption and extends flight endurance, which is particularly critical for UAV platforms with limited battery capacity. Its consistently high path feasibility and low variance ensure safe navigation in environments containing obstacles, threat zones, or complex terrain, thereby enhancing mission reliability. These characteristics highlight the algorithm’s capability to balance solution quality, operational safety, and computational efficiency. Although CTWRBMO demonstrates strong performance under the current experimental conditions, its applicability to multi-objective optimization tasks and real-time path planning requires further investigation. Future research will involve in-depth testing in actual flight environments to comprehensively evaluate its engineering feasibility and robustness in practical applications.

## Figures and Tables

**Table 1:** Comparison of the mathematical structures of RBMO and CTWRBMO.

Module	Original RBMO	CTWRBMO	Structural Difference
Initialization strategy	Linear random distribution	Circle map (Equation (7))	Random → chaotic
Search probability ε	Constant ε=0.5	$\varepsilon (t)=c\times \left(1-\left(\frac{t}{T}\right)\right)$ (Equation (8))	Fixed → adaptive
Disturbance weight wd	None	$\mathrm{Exponential}\;\mathrm{decay}\;wd\left(t\right)$ (Equation (9))	Fixed → shrinking
Position update operator	Global-best only	Neighborhood differential update (Equations (10)–(14))	Single-source → multi-source
Elite strategy	None	Greedy acceptance + neighborhood elite retention	No elite → preserved elite

**Table 2:** Algorithm parameter settings.

Arithmetic	Parameterization
PSO	$\omega_{\max}=0.9,\omega_{\min}=0.2,\;c_{1}=c_{2}=2$
HHO	$E_{1}=2\times \left(1-\frac{t}{T}\right),E_{0}\in \left(-1,1\right)$
GTO	p=0.03, β=3, ω=0.8
AVOA	$p_{1}=0.6,p_{2}=0.4,p_{3}=0.6,\;\alpha =0.8,\beta =0.2,\gamma =2.5$
RBMO	ε=0.5
GWO	$a=2-\frac{2t}{T}$
L-SHADE	$memory_{size}=5,\;{pbest}_{size}=5,\;H=6$
WOA	$a=2-\frac{2t}{T},\;a_{2}=-1+t\cdot \frac{-1}{T}$
CTWRBMO	$\varepsilon =0.62\times \left(1-\left(\frac{t}{T}\right)\right)$

**Table 3:** CEC2020 test functions.

	No.	Functions	$F_{i}^{*}=F_{i}(x^{*})$
Unimodal Function	1	Shifted and Rotated Bent Cigar Function	100
Basic Functions	2	Shifted and Rotated Schwefel’s Function	1100
	3	Shifted and Rotated Lunacek bi-Rastrigin Function	700
	4	Expanded Rosenbrock’s plus Griewangk’s Function	1900
Hybrid Functions	5	Hybrid Function 1 (N = 3)	1700
	6	Hybrid Function 2 (N = 4)	1600
	7	Hybrid Function 3 (N = 5)	2100
Composition Functions	8	Composition Function 1 (N = 3)	2200
	9	Composition Function 2 (N = 4)	2400
	10	Composition Function 3 (N = 5)	2500
$\mathrm{Search}\;\mathrm{range}:\;{[-100,100]}^{D}$			

**Table 4:** Optimization values of algorithms.

		PSO	L-SHADE	HHO	WOA	GTO	GWO	AVOA	RBMO	CTWRBMO
F1	best	101.0841	5.65 × 10^3^	7.97 × 10^6^	3.44 × 10^8^	106.9128	2.12 × 10^5^	122.5997	135.4385	**100.4225**
	std	1.34 × 10^9^	9.25 × 10^6^	2.47 × 10^7^	7.12 × 10^8^	3.66 × 10^3^	1.17 × 10^9^	4.35 × 10^3^	4.05 × 10^3^	**2.18 × 10^3^**
	mean	6.39 × 10^8^	8.46 × 10^7^	3.70 × 10^7^	1.07 × 10^9^	7.37 × 10^3^	5.36 × 10^5^	4.00 × 10^3^	4.50 × 10^3^	**2.14 × 10^3^**
F2	best	**1.46 × 10^3^**	2.69 × 10^3^	2.68 × 10^3^	2.90 × 10^3^	2.53 × 10^3^	2.03 × 10^3^	1.9 × 10^3^	2.06 × 10^3^	1.75 × 10^3^
	std	**412.9828**	857.5273	490.816	516.1092	616.7485	569.8171	550.2542	484.739	465.9739
	mean	2.15 × 10^3^	2.74 × 10^3^	3.85 × 10^3^	4.70 × 10^3^	3.33 × 10^3^	2.46 × 10^3^	2.94 × 10^3^	3.04 × 10^3^	**1.88 × 10^3^**
F3	best	736.1379	736.2967	866.5471	897.6801	776.8541	761.9352	810.1968	735.7967	**729.6288**
	std	13.5036	31.2346	35.7475	50.1808	40.1867	18.6996	37.5011	13.9754	**9.775**
	mean	766.0323	786.193	906.4648	957.578	884.8169	799.5011	876.952	767.1871	**751.6675**
F4	best	1.90 × 10^3^	1.90 × 10^3^	1.90 × 10^3^	1.90 × 10^3^	1.90 × 10^3^	1.90 × 10^3^	1.90 × 10^3^	1.90 × 10^3^	**1.90 × 10^3^**
	std	1.37 × 10^1^	22.0152	16.7124	846.2364	23.1039	62.0629	8.111	1.3444	**0.8697**
	mean	1.91 × 10^3^	1.91 × 10^3^	1.93 × 10^3^	2.21 × 10^3^	1.92 × 10^3^	1.91 × 10^3^	1.92 × 10^3^	1.90 × 10^3^	**1.90 × 10^3^**
F5	best	1.12 × 10^4^	8.10 × 10^3^	4.89 × 10^4^	5.22 × 10^5^	5.07 × 10^3^	2.53 × 10^4^	2.23 × 10^4^	2.12 × 10^3^	**1.96 × 10^3^**
	std	3.06 × 10^5^	7.50 × 10^5^	5.82 × 10^5^	2.36 × 10^6^	3.99 × 10^4^	1.05 × 10^6^	6.49 × 10^5^	243.4966	**242.7868**
	mean	3.15 × 10^5^	7.13 × 10^5^	5.40 × 10^5^	2.25 × 10^5^	5.77 × 10^4^	2.32 × 10^6^	1.35 × 10^5^	**2.47 × 10^3^**	2.49 × 10^3^
F6	best	1.62 × 10^3^	1.60 × 10^3^	1.84 × 10^3^	2.17 × 10^3^	1.64 × 10^3^	1.64 × 10^3^	1.64 × 10^3^	1.63 × 10^3^	**1.60 × 10^3^**
	std	164.0235	164.8115	280.1947	287.448	207.7368	216.797	227.9112	187.0467	**148.5156**
	mean	1.79 × 10^3^	**1.66 × 10^3^**	2.57 × 10^3^	2.35 × 10^3^	2.57 × 10^3^	1.89 × 10^3^	1.83 × 10^3^	1.89 × 10^3^	1.74 × 10^3^
F7	best	1.15 × 10^4^	2.97 × 10^3^	1.34 × 10^4^	9.79 × 10^4^	3.10 × 10^3^	1.94 × 10^4^	1.91 × 10^4^	2.39 × 10^3^	**2.21 × 10^3^**
	std	2.86 × 10^5^	1.39 × 10^5^	7.07 × 10^5^	2.08 × 10^6^	7.49 × 10^3^	4.99 × 10^3^	4.97 × 10^5^	189.6367	**159.9077**
	mean	4.73 × 10^4^	3.04 × 10^4^	1.98 × 10^5^	5.21 × 10^6^	6.39 × 10^3^	7.62 × 10^4^	6.11 × 10^5^	2.69 × 10^3^	**2.61 × 10^3^**
F8	best	2.30 × 10^3^	2.30 × 10^3^	2.32 × 10^3^	2.37 × 10^3^	2.30 × 10^3^	2.32 × 10^3^	2.30 × 10^3^	2.30 × 10^3^	**2.30 × 10^3^**
	std	890.972	1.93 × 10^3^	1.67 × 10^3^	1.84 × 10^3^	**2.0761** × 10^3^	1.39 × 10^3^	1.30 × 10^3^	961.653	34.7868
	mean	2.37 × 10^3^	6.56 × 10^3^	3.69 × 10^3^	7.04 × 10^3^	2.30 × 10^3^	4.39 × 10^3^	2.31 × 10^3^	3.95 × 10^3^	**2.30 × 10^3^**
F9	best	2.86 × 10^3^	2.84 × 10^3^	2.91 × 10^3^	2.93 × 10^3^	2.85 × 10^3^	2.82 × 10^3^	2.85 × 10^3^	2.83 × 10^3^	**2.50 × 10^3^**
	std	47.6091	24.449	112.5738	70.6194	45.1452	32.9734	66.355	18.5983	**17.7293**
	mean	2.95 × 10^3^	2.93 × 10^3^	3.07 × 10^3^	3.12 × 10^3^	2.94 × 10^3^	2.96 × 10^3^	3.07 × 10^3^	2.86 × 10^3^	**2.83 × 10^3^**
F10	best	2.91 × 10^3^	2.91 × 10^3^	2.94 × 10^3^	2.98 × 10^3^	2.91 × 10^3^	2.95 × 10^3^	2.91 × 10^3^	2.91 × 10^3^	**2.90 × 10^3^**
	std	50.6296	**25.6281**	34.5923	55.0825	31.8364	58.0418	38.8186	30.2213	35.1898
	mean	2.94 × 10^3^	**2.91 × 10^3^**	2.99 × 10^3^	3.14 × 10^3^	2.97 × 10^3^	3.01 × 10^3^	3.02 × 10^3^	2.95 × 10^3^	2.92 × 10^3^

**Table 5:** Wilcoxon rank-sum test *p*-values.

Algorithm	PSO	LSHADE	HHO	WOA	GTO	GWO	AVOA	RBMO
F1	**3.01 × 10^−7^**	**1.87 × 10** **^−^** **^7^**	**3.02 × 10** **^−^** **^11^**	**3.02 × 10** **^−^** **^11^**	6.52 × 10^−1^	**3.0199 × 10** **^−^** **^11^**	1.91 × 10^−1^	**4.06 × 10** **^−^** **^2^**
F2	**4.90 × 10** **^−^** **^3^**	**2.92 × 10** **^−^** **^9^**	**2.78 × 10** **^−^** **^7^**	**1.21 × 10** **^−^** **^10^**	**1.53 × 10** **^−^** **^5^**	**8.77 × 10** **^−^** **^2^**	**1.12 × 10** **^−^** **^2^**	**1.08 × 10** **^−^** **^2^**
F3	**5.35 × 10** **^−^** **^1^**	**2.25 × 10** **^−^** **^4^**	**3.02 × 10** **^−^** **^11^**	**3.02 × 10** **^−^** **^11^**	**8.9934 × 10** **^−^** **^11^**	**2.6784 × 10** **^−^** **^6^**	**3.0199 × 10** **^−^** **^11^**	**2.17 × 10** **^−^** **^1^**
F4	**3.5201 × 10** **^−^** **^7^**	**3.35 × 10** **^−^** **^8^**	**3.02 × 10** **^−^** **^11^**	**3.02 × 10** **^−^** **^11^**	**3.6897 × 10** **^−^** **^11^**	**3.3384 × 10** **^−^** **^11^**	**3.0199 × 10** **^−^** **^11^**	2.30 × 10^−1^
F5	**3.0199 × 10** **^−^** **^11^**	**3.02 × 10** **^−^** **^11^**	**3.02 × 10** **^−^** **^11^**	**3.02 × 10** **^−^** **^11^**	**6.6955 × 10** **^−^** **^11^**	**3.02 × 10** **^−^** **^11^**	**3.02 × 10** **^−^** **^11^**	**1.44 × 10** **^−^** **^2^**
F6	**2.15 × 10** **^−^** **^2^**	**1.38 × 10** **^−^** **^2^**	**1.17 × 10** **^−^** **^9^**	**3.82 × 10** **^−^** **^10^**	**4.01 × 10** **^−^** **^2^**	**1.08 × 10** **^−^** **^2^**	**3.50 × 10** **^−^** **^3^**	**3.03 × 10** **^−^** **^2^**
F7	**3.0199 × 10** **^−^** **^11^**	**3.02 × 10** **^−^** **^11^**	**3.02 × 10** **^−^** **^11^**	**3.02 × 10** **^−^** **^11^**	**1.21 × 10** **^−^** **^11^**	**3.02 × 10** **^−^** **^11^**	**3.0199 × 10** **^−^** **^11^**	**7.30 × 10** **^−^** **^3^**
F8	**7.30 × 10** **^−^** **^3^**	**9.10 × 10** **^−^** **^3^**	**1.00 × 10** **^−^** **^3^**	**3.30 × 10** **^−^** **^3^**	**3.45 × 10** **^−^** **^1^**	**1.70 × 10** **^−^** **^3^**	2.41 × 10^−1^	**4.20 × 10** **^−^** **^3^**
F9	**1.21 × 10** **^−^** **^10^**	**2.15 × 10** **^−^** **^10^**	**3.02 × 10** **^−^** **^11^**	**3.02 × 10** **^−^** **^11^**	**1.01 × 10** **^−^** **^8^**	**5.94 × 10** **^−^** **^2^**	**2.87 × 10** **^−^** **^10^**	**1.54 × 10** **^−^** **^1^**
F10	**1.50 × 10** **^−^** **^1^**	**1.00 × 10** **^−^** **^3^**	**3.30 × 10** **^−^** **^4^**	**1.83 × 10** **^−^** **^4^**	**3.60 × 10** **^−^** **^3^**	**0.0013**	**4.60 × 10** **^−^** **^3^**	**1.50 × 10** **^−^** **^2^**
	**8/10**	**10/10**	**10/10**	**10/10**	**8/10**	**8/10**	**8/10**	**7/10**

**Table 6:** Comparison of ablation results of the test functions under different improvement strategies.

Algorithm		RBMO	IRBMO1	IRBMO2	IRBMO3	IRBMO4	CTWRBMO
F2	best	1.94 × 10^3^	1.69 × 10^3^	1.94 × 10^3^	1.93 × 10^3^	1.77 × 10^3^	**1.69 × 10^3^**
	std	517.6349	496.1124	402.2417	450.2726	502.5877	**395.8793**
	mean	2.90 × 10^3^	2.29 × 10^3^	2.79 × 10^5^	2.75 × 10^3^	2.29 × 10^3^	**2.12 × 10^3^**
F3	best	737.159	736.2486	736.711	736.6805	733.8313	**732.3325**
	std	13.7076	12.2874	11.7825	11.2594	12.9146	**10.9219**
	mean	769.6035	745.2775	759.5666	752.82	746.302	**740.5662**
F4	best	**1.90 × 10^3^**	**1.90 × 10^3^**	**1.90 × 10^3^**	**1.90 × 10^3^**	**1.90 × 10^3^**	**1.90 × 10^3^**
	std	1.0707	1.0237	1.046	1.022	**0.6787**	0.9681
	mean	1.91 × 10^3^	1.90 × 10^3^	1.90 × 10^3^	1.91 × 10^3^	1.90 × 10^3^	**1.90 × 10^3^**
F7	best	2.40 × 10^3^	2.28 × 10^3^	2.37 × 10^3^	2.37 × 10^3^	2.36 × 10^3^	**2.28 × 10^3^**
	std	217.7298	170.9036	**158.3269**	173.2306	179.5852	164.0942
	mean	2.85 × 10^3^	2.52 × 10^3^	2.55 × 10^3^	2.57 × 10^3^	2.68 × 10^3^	**2.49 × 10^3^**
F9	best	2.84 × 10^3^	2.82 × 10^3^	2.83 × 10^3^	2.84 × 10^3^	2.8 × 10^3^	**2.82**
	std	20.9272	16.6296	15.813	14.9577	16.8652	**11.4647**
	mean	2.87 × 10^3^	2.83 × 10^3^	2.84 × 10^3^	2.85 × 10^3^	2.83 × 10^3^	**2.82 × 10^3^**
F10	best	2.91 × 10^3^	2.90 × 10^3^	2.91 × 10^3^	2.91 × 10^3^	2.90 × 10^3^	**2.90 × 10^3^**
	std	36.4438	25.6816	31.169	28.8592	28.1338	**24.637**
	mean	2.97 × 10^3^	2.96 × 10^3^	2.94 × 10^3^	2.91 × 10^3^	2.95 × 10^3^	**2.91 × 10^3^**
+/=/−		**/**	**17/1/0**	**17/1/0**	**17/1/0**	**17/1/0**	**17/1/0**

**Table 7:** Terrain parameters.

Mountain Model	Environmental Limits	Start Point	Target Point	Number of Threats
Environment 1	500 × 500 × 500	15.15, 30.3, 295.9	449.5, 459.6, 422	3
Environment 2	500 × 500 × 500	15.15, 30.3, 295.9	449.5, 459.6, 422	6

**Table 8:** Threat Model Parameters.

Mountain Mode	Threat ID	Threat Center (X, Y)	Threat Radius R	Threat Height H
**Environment 1**	1	207.1, 333.3	30	389.9
	2	393.9, 414.1	50	349.2
	3	423.0, 324.0	50	350.0
**Environment 2**	1	367.1, 237.3	40	339.9
	2	393.9, 414.1	50	349.2
	3	423.0, 324.0	50	350.0
	4	292.2, 358.4	50	350.6
	5	206.8, 276.1	30	300.2
	6	242.4, 151.5	50	313.1

**Table 9:** Algorithm parameter settings.

Arithmetic	Parameterization
ABC	$\omega_{\max}=0.9,\omega_{\min}=0.2,c_{1}=c_{2}=2$
HHO	$\mathrm{Food}\;\mathrm{Number}=\frac{\mathrm{NP}}{2}$
AVOA	$p_{1}=0.6,p_{2}=0.4,p_{3}=0.6,\alpha =0.8,\beta =0.2,\gamma =2.5$
RBMO	ε=0.5
CTERBMO	$\varepsilon =0.62\times \left(1-\left(\frac{t}{T}\right)\right)$

**Table 10:** Path planning performance metrics of different algorithms.

Mountain Model	Indicators	CTWRBMO	RBMO	HHO	ABC	AVOA
Environment 1	Optimal path length	**749.21**	783.19	999.22	757.82	998.4
	Average path cost	**751.171**	799.452	1177.95	784.885	1095.25
	Variance	**0.57**	18.54	92.66	24.9	59.67
	Average computation time	423.7117 s	443.3562 s	582.2019 s	392.26 s	**395.644 s**
	Average smoothness	**0.0251 rad**	0.0314 rad	0.0316 rad	0.0267 rad	0.027 rad
	Average energy consumption	**809.27**	965.6	1150.86	1042.37	1225.6
	Success rate	**1**	**1**	0.53	**1**	0.67
Environment 2	Optimal path length	**751.38**	789.87	946.11	939.93	952.06
	Average path cost	**751.77**	802.216	1077.4	801.251	1095.5
	Variance	**0.31**	19.61	92.94	75.07	97.55
	Average computation time	426.627 s	451.3562 s	584.6893 s	396.75 s	**397.327 s**
	Average smoothness	**0.0256 rad**	0.0264 rad	0.0311 rad	0.0324 rad	0.0284 rad
	Average energy consumption	**809.34**	1088.63	1361.5	1097.89	1246.92
	Success rate	**1**	0.93	0.40	**1**	0.63

## Data Availability

The original contributions presented in this study are included in the article.
